# A Year at the Forefront of Gliding Locomotion

**DOI:** 10.1242/bio.059973

**Published:** 2023-08-15

**Authors:** Pranav C. Khandelwal, Mohamed A. Zakaria, John J. Socha

**Affiliations:** ^1^ Max Planck Institute for Intelligent Systems, Stuttgart 70569, Germany; ^2^ Institute of Flight Mechanics and Controls, University of Stuttgart, Stuttgart 70569, Germany; ^3^ Department of Aerospace and Ocean Engineering, Virginia Tech, Blacksburg, VA 24061, USA; ^4^ Department of Biomedical Engineering and Mechanics, Virginia Tech, Blacksburg, VA 24061, USA

**Keywords:** Animals, Aquatic, Flapping, Non-flapping, Hypothesis

## Abstract

This review highlights the largely understudied behavior of gliding locomotion, which is exhibited by a diverse range of animals spanning vertebrates and invertebrates, in air and in water. The insights in the literature gained from January 2022 to December 2022 continue to challenge the previously held notion of gliding as a relatively simple form of locomotion. Using advances in field/lab data collection and computation, the highlighted studies cover gliding in animals including seabirds, flying lizards, flying snakes, geckos, dragonflies, damselflies, and dolphins. Altogether, these studies present gliding as a sophisticated behavior resulting from the interdependent aspects of morphology, sensing, environment, and likely selective pressures. This review uses these insights as inspiration to encourage researchers to revisit gliding locomotion, both in the animal's natural habitat and in the laboratory, and to investigate questions spanning gliding biomechanics, ecology, sensing, and the evolution of animal flight.

## Teaser

**Summary:** This review further challenges the previous notion of gliding as a relatively simple form of aerial locomotion. It reinforces the concept of gliding as a sophisticated behavior that involves the interdependent aspects of morphology, sensing, environment, and likely selective pressures.

## Introduction

**Fig. 1.**
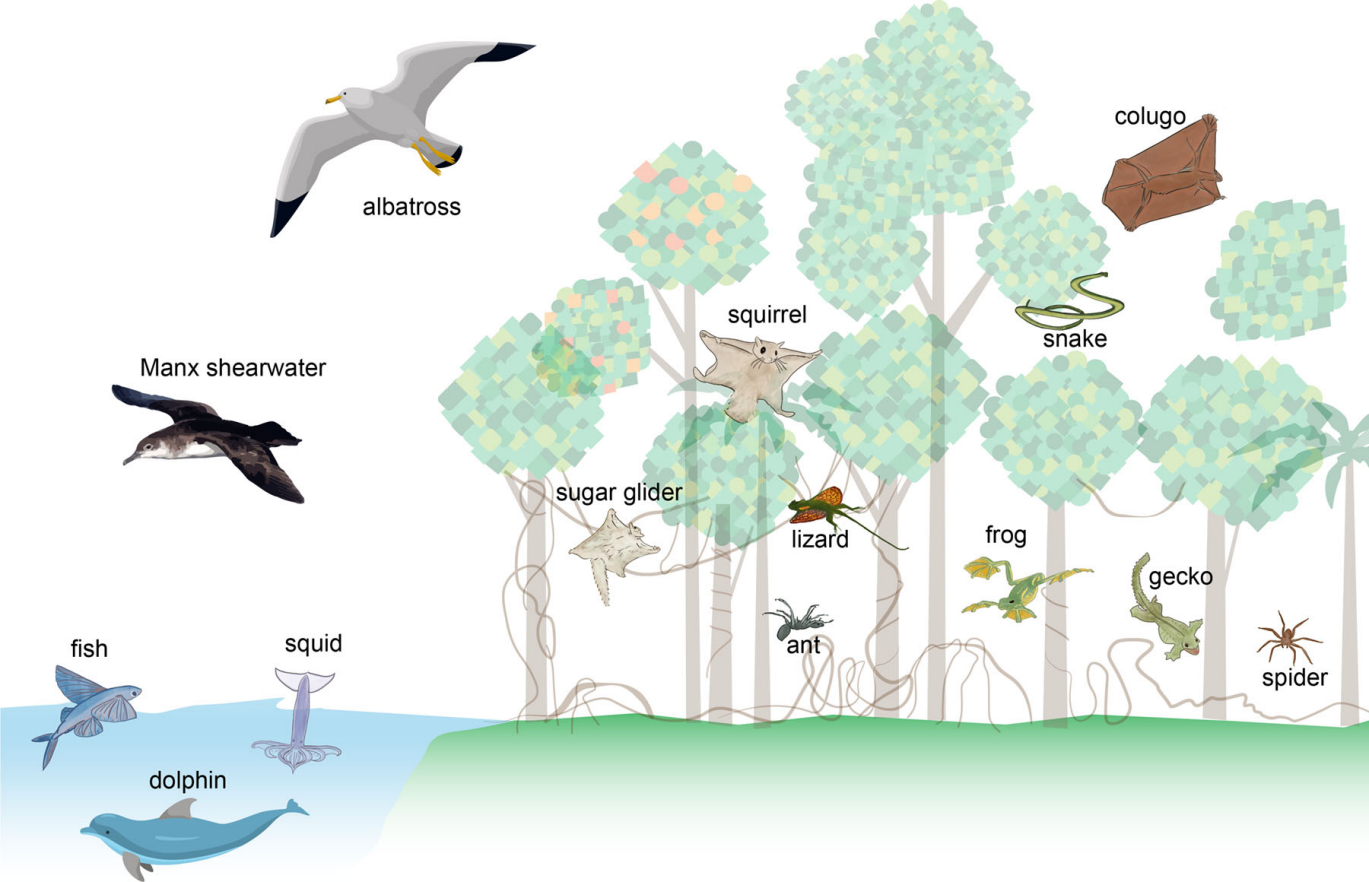
**A diversity of flapping and non-flapping flyers use gliding locomotion to carry out their day-to-day activities.** Illustration not to scale and adapted from (Khandelwal et al., 2023). Image credits: non-flapping flyer illustrations, Dr Mary K. Salcedo; albatross, Svitlana/Adobe Stock; dolphin, bigmouse108/Adobe Stock; Manx shearwater, tonymills/Adobe Stock.

A broad range of taxa employ gliding, a fluid-based form of locomotion in which the animal moves downward and horizontally without generating thrust (Dudley et al., 2007; Khandelwal et al., 2023; Socha et al., 2015). Instead, the animal trades potential energy for kinetic energy and uses its velocity to generate forces of lift and drag. These forces counteract the pull of gravity and produce horizontal movement. Many birds, bats, and insects primarily flap their wings to power flight, but some of these species can also hold their wings steady to glide and descend gently in air (Bomphrey et al., 2016; Thomas et al., 1990; Tobalske, 2001). All other flyers cannot flap to generate thrust and rely solely on gravity to move aerially in their natural habitat; these non-flapping flyers are also known as gliders (Khandelwal et al., 2023; Socha et al., 2015). Nearly all gliders possess body modifications in conjunction with behavioral adaptations that allow them to produce and control aerodynamic forces for horizontal movement during aerial descent (Khandelwal et al., 2023; Socha et al., 2015). For example, flying lizards and gliding mammals can actively modify their body parts to act like wings, snakes can flatten their body to increase surface area, and gliding ants possess flattened limbs that increase their body surface area compared to their non-gliding counterparts (Yanoviak et al., 2010; Khandelwal et al., 2023; Socha et al., 2014). Gliding as part of intermittent locomotion is also employed by aquatic animals underwater (Williams, 2001). Periods of burst/stroke are followed by gliding, which is associated with lower costs of locomotion (Floryan et al., 2017; Kramer and McLaughlin, 2001; Xia et al., 2018). Seals, sharks, and dolphins are a few examples of aquatic animals that use gliding to move in water. Fig. 1 provides an illustration of a subset of animals that use gliding to move in their natural habitat.

The conception of the animal holding a steady pose during gliding has often led to the assumption that gliding is a relatively simple form of locomotion. However, studies in recent years have challenged this notion: gliding has emerged as a sophisticated locomotor mode that integrates morphology, sensing, behavior, and judgment of the animal. Advances in understanding the mechanics of gliding has driven the shift from models of the animal as a rigid, fixed-wing craft to dynamic systems that incorporate the effects of active changes in body shape and size, which have a non-trivial impact on the physics of gliding locomotion (Harvey and Inman, 2022; Khandelwal and Hedrick, 2022; Lentink et al., 2007; Yeaton et al., 2020). The capability of the animal to sense and judge its surroundings to glide has been shown to be extremely important for flapping and non-flapping flyers alike. For example, flapping flyers need to precisely sense upward drafts to soar and glide for extended durations of time (Kempton et al., 2022). Non-flapping flyers, in the absence of thrust generation, have to sense and judge their surroundings to plan their glide trajectory, negotiate obstacles, and identify landing targets (Khandelwal and Hedrick, 2020; Khandelwal et al., 2023).

Much of our new understanding of gliding locomotion has been made possible by advances in experimental and computational methods. Techniques including motion capture and on-body sensing have significantly expanded the scope of field studies on gliding, leading to discoveries in gliding capabilities, behavior, and sensing in animals. Computational fluid dynamics (CFD), particle image/tracking velocimetry (PIV/PTV), and wind tunnel testing have provided granular details on the physics of gliding, highlighting the importance of morphology and body control.

Overall, studies continue to probe questions on the physics, behavior, and sensing involved during gliding to provide a more holistic understanding of gliding locomotion in animals. Here, we highlight studies on gliding locomotion in the period January 2022 to December 2022 (Table 1). These studies represent a subset of the advancements made in this area and help envision the future prospects of research on this sophisticated yet tractable mode of locomotion.

**Table 1.**
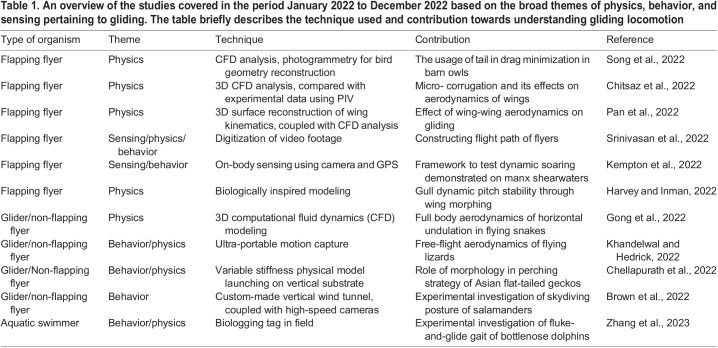
An overview of the studies covered in the period January 2022 to December 2022 based on the broad themes of physics, behavior, and sensing pertaining to gliding. The table briefly describes the technique used and contribution towards understanding gliding locomotion

## Non-flapping flyers

**Fig. 2.**
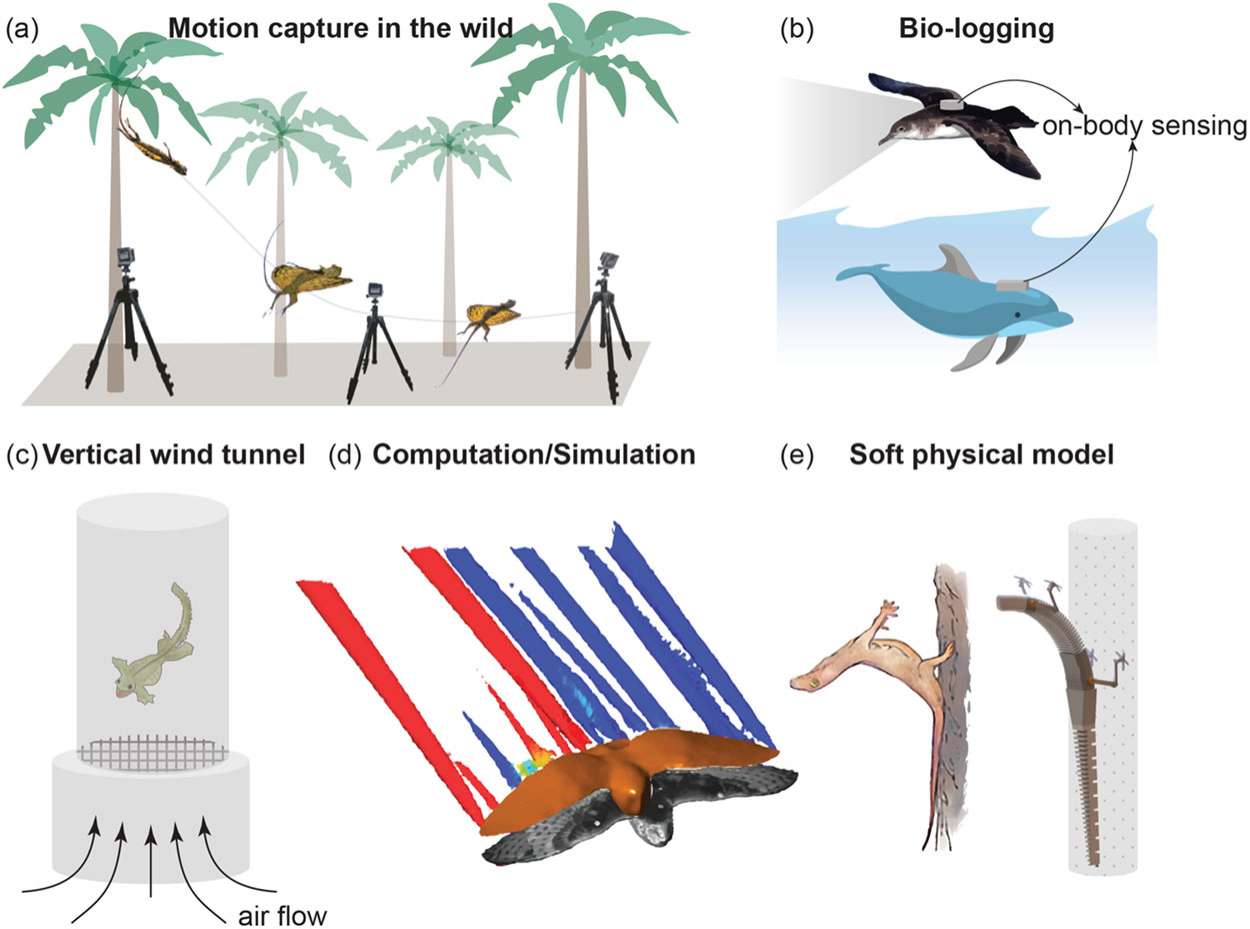
**An overview of technological innovations leading to novel insights on gliding locomotion.** (A) An ultra-portable motion capture system that allows the study of gliding behavior/biomechanics in the wild. In Khandelwal and Hedrick (2022) it was used to quantify the free-flight aerodynamics of flying lizards. (B) Bio-logging allows for position and kinematic data collection over extended durations of time and during natural behavior. Kempton and colleagues (2022) used a video logger and GPS device on the Manx shearwater to collect the bird's pitch angle, bank angle, and position. In Zhang et al., 2023, a custom-made bio-logger was used that included an accelerometer, gyroscope, magnetometer, pressure sensor, temperature sensor, and speed sensor to quantify the fluke-glide dynamics of bottlenose dolphins. (C) A vertical wind tunnel allows controlled testing of gliding biomechanics in non-flapping flyers. A vertical wind tunnel was used to show that wandering salamanders hold a skydiving pose to glide (Brown et al., 2022). For illustration, here we show a gliding gecko instead. (D) A combination of particle tracking velocimetry (PTV) and computational fluid dynamics (CFD) facilitate a more granular understanding of the physics of gliding flight. CFD simulations agreed well with PTV to show that specific tail poses reduce drag while gliding (adapted from Song et al., 2022). (E) Soft physical models enable the investigation of the effects of morphology and the underlying movement control on gliding locomotion. Chellapurath and colleagues (2022) used a soft physical model to show the importance of torso and tail stiffness in gliding geckos to successfully perch on vertical substrates in the absence of enlarged aerodynamic surfaces (adapted from Chellapurath et al., 2022). 3D rendering of the soft physical model of the gecko by Dr Mrudul Chellapurath.

The year in review saw the discovery of aerial capabilities in wandering salamanders (*Aneides vagrans*). Brown and colleagues (Brown et al., 2022) showed that salamanders use a skydiving pose to hold position in a vertical wind tunnel, enabling parachuting from trees in their native environment (Fig. 2C). The salamanders also exhibited body/tail undulations that were correlated with horizontal movement in the tunnel, which suggests that they could undertake steep glides using this mechanism. This discovery adds another species to our understanding of the diversity of non-flapping animals that have independently evolved gliding, a functional group that include mammals, snakes, lizards, frogs, insects, spiders, fish, and squid. New insights were also gained in well-known gliders like the flying lizard and flying snake. Khandelwal and Hedrick have demonstrated the use of an aerodynamic strategy in flying lizards (*Draco dussumieri*) that maximizes their glide distance while allowing them to modulate the aerodynamic force production through changes in body shape and posture (Khandelwal and Hedrick, 2022). Furthermore, these lizards were able to maintain lift generation even at angles of incidence greater than 55° between their wing-like body and oncoming air (angle of attack, AoA), showing the aerodynamic advantages of their body and wing design at high AoA.

Unlike flying lizards, flying snakes do not have a dedicated wing membrane and flatten their body to form a wing, making conventional approaches to aerodynamic analysis challenging. Gong and colleagues (Gong et al., 2022) used 3D computational modeling to study the flow around a flying snake undergoing horizontal undulation during gliding, providing the first insight into the unsteady, full-body aerodynamics of this glider. By contrast, recent work (Yeaton et al., 2020) examined the mechanics of the full body using 2D coefficients and quasi-static assumptions. Interestingly, the new study finds the maximum lift-to-drag ratio of the snake-like airfoil at ∼20°, which is comparable to the AoA at which flying lizards, with a drastically different airfoil design, achieve their maximum lift-to-drag ratio. This result contradicts previous aerodynamic studies that show a maximum at a greater AoA of 35° (Holden et al., 2014; Krishnan et al., 2014; Miklasz et al., 2010), suggesting that increased fidelity of modeling is important for understanding gliding aerodynamics. More sophisticated modeling that incorporates vertical movement and other aspects of morphology (such as the tail) is needed to resolve this question.

## Flapping flyers

A field study of Manx shearwaters (*Puffinus puffinus*) showed new empirical evidence of dynamic soaring in seabirds, a specialized form of gliding that was previously empirically shown only in albatross (Kempton et al., 2022). In doing so, the study provides a framework to test dynamic soaring in other bird species that employ intermittent flapping flight in coastal environments. Beyond the field discovery, a new role of bird tails as a drag-reduction device while gliding was shown in barn owls (*Tyto alba*) (Song et al., 2022); previously, bird tails were largely considered as a steering device during gliding. The numerical results of Song and colleagues (Song et al., 2022) were consistent with observations in live gliding owls (Fig. 2D). Both examinations showed that spreading the tail and pointing it downwards maintains the tail's contribution of supporting body weight while also reducing the overall drag. In another study, Harvey and Inman (Harvey and Inman, 2022) used modeling to show how gulls control their pitch stability via wing morphing during gliding. Their study demonstrates the importance of incorporating biological information in conventional aerodynamic modeling and demonstrates a mechanism by which birds can shift between stable flight and agile maneuvers.

Investigations on insect gliding have shown the advantages of wing corrugation to delay flow separation and increase the lift-to-drag ratio compared to flat wings. However, these insights have been garnered mostly using 2D models of the cross-section of the wing, which do not capture substantial variation in wing corrugation across the insect wingspan. Chitsaz and colleagues used a combination of CFD and PIV on a complete 3D dragonfly (*Orthetrum caledonicum*) wing model to show the entrapment of vortices in the valleys of wing corrugation and, in doing so, provide an illustrative example of how to analyze flow characteristics in other corrugated wing designs (Chitsaz et al., 2022). Along with corrugation, insects such as dragonflies and damselflies also feature tandem wings, with both a fore- and hindwing. Pan and colleagues examined the fore- and hindwing configuration observed during natural gliding flight in damselflies, and used CFD to demonstrate that this configuration might enhance overall gliding performance: that the gliding performance of the forewing, when tested with the hindwing, was improved compared to its solitary performance (Pan et al., 2022).

## Underwater gliding

The use of gliding underwater along with fluking (undulatory propulsive motion of the tail fin) is a component of intermittent locomotion performed by some aquatic animals including sharks, seals, and dolphins to lower their cost of transport. Zhang and colleagues developed a fluke-glide model for bottlenose dolphins and estimated drag, energetic efficiency, and gait dynamics (Zhang et al., 2023). The underlying kinematic data used to develop the model were collected from freely-swimming dolphins in a large aquarium tank (Fig. 2B). The dolphins voluntarily switched between fluke and glide gaits, which provided a more biologically relevant input compared to previous studies that used structured trials. Their results showed significant energy savings during fluke-glide compared to fluke-only swimming mode in dolphins for a given speed.

## Technological innovations

### Motion capture

Studies on gliding locomotion have benefitted from advancements in engineering and technology. Khandelwal and Hedrick constructed an ultra-portable motion capture setup using off-the-shelf cameras to perform markerless multi-point tracking of flying lizards (∼10 cm in body size) over a glide distance of 5.5 m in the animals’ natural habitat (Khandelwal and Hedrick, 2022). The setup demonstrated an inexpensive way to collect 3D biomechanics data in the wild with lab-like fidelity while being more robust and rugged compared to expensive high-speed cameras (Fig. 2A).

The possibility of obtaining 3D data from a single camera for biomechanical studies was demonstrated by Srinivasan and colleagues (Srinivasan et al., 2022). Using a single camera, the authors were able to track the flight trajectory of birds (budgerigars, *Melopsittacus undulatus*) through an indoor tunnel in three dimensions. However, the approach requires prior knowledge of one or more body measurements of the animal to measure actual distances, but body dimension information is not necessary for relative measurements. The technique provides a cost-effective approach to studying gliding flight, but might be prone to errors in tracking absolute changes in body shape and size.

### Computational techniques

Advancements in 3D scanning and photogrammetry have allowed more accurate representations of the animal's gliding apparatus, consequently leading to more biologically relevant physical interpretations using CFD and numeric simulations. Pan and colleagues and Chitsaz and colleagues show examples of new insights on aerodynamics of gliding in dragonflies and damselflies that were gained through more accurate representations of the wing structure and their glide behavior (Pan et al., 2022; Chitsaz et al., 2022).

### Bioinspired soft physical models

The bodies of animals are soft and compliant structures that contribute to their sophisticated movements. Advances in robotics have enabled the development of physical platforms that can replicate some of these movements. These platforms act as powerful tools to interact with real-world surroundings and in doing so, serve as a test bed to explore parameters, inform models, and test various hypotheses (an approach also known as ‘robophysics’; Aguilar et al., 2016). Chellapurath and colleagues used a simple and inexpensive soft physical model to replicate the perching behavior of gliding geckos, which is extremely challenging to elicit in live specimens (Chellapurath et al., 2022; Fig. 2E). Their results demonstrated the critical role of a compliant torso and tail to perch in the absence of aerodynamic control surfaces used in some other gliding species to slow their descent during landing.

## New gliding datasets

Studies on gliding locomotion have mostly focused on either modeling or live animal experiments. Modeling studies often lack biological data to tune the model parameters and validate the results. Live animal studies are often experimentally and ethically constrained, and thereby can benefit from modeling. Open-source data and models can help bridge the gap between the two. Khandelwal and Hedrick (2022) provide kinematic, body pose, and aerodynamic data on flying lizards, which can be used to inform modeling studies and act as a comparative dataset for studies on other gliding taxa.

## New hypotheses

The year in review has provided new insights on gliding locomotion and shown the possibility of investigating hypotheses related to biomechanics, ecology, and sensing that were not previously possible. Here, we present a few hypotheses and directions of investigation that we hope will spur further development of testable hypotheses on gliding locomotion.

### Non-flapping flyers

#### Path-planning strategy

Khandelwal and Hedrick (2020) showed that in the absence of thrust-generation capabilities, flying lizards use a path-planning strategy to account for obstacles and glide distance to reach their desired target. Such a strategy is favorable to reduce energetic losses due to reactive maneuvering, and it also increases the chances of reaching the glide target. A similar strategy is likely prevalent in other terrestrial gliding animals that inhabit spatially cluttered habitats, a target for future study. Furthermore, the degree of aerodynamic control exhibited by gliders varies based on the type of aerodynamic surfaces they possess; mammalian gliders and flying lizards have well-defined wing-like surfaces that provide greater aerodynamic control compared to other gliders (Khandelwal et al., 2023). Therefore, the relative importance of a path-planning strategy can vary across gliding taxa; animals with less aerodynamic control (e.g., ants, geckos, frogs, snakes) might experience higher selective pressures to employ a path-planning strategy than mammalian gliders and flying lizards. A comparative study is required to test this hypothesis.

#### Maximizing glide distance

To conserve energy during horizontal transport, non-flapping flyers should maximize their glide distance, which they can achieve by operating at their maximal lift-to-drag ratio resulting in a shallower glide. Khandelwal and Hedrick (2022) empirically showed that flying lizards glide at their near-maximal lift-to-drag ratio, providing preliminary support for the hypothesis of maximizing glide distance in this species. This data from the field is corroborated by recent modeling results (Lau et al., 2023), which also show high lift-to-drag ratios at the angles of attack exhibited by the real animals. A similar hypothesis remains to be explored in other non-flapping flyers, but requires live animal data that captures the kinematics as well as the body pose of the animal in free flight.

#### Body control

A successful glide involves the animal changing its body shape, size, and appendage position to produce aerodynamic and inertial forces conducive for its desired aerial behavior (e.g., takeoff, maneuvering, and landing). Recent studies on flying lizards (Khandelwal and Hedrick, 2022) and flying snakes (Gong et al., 2022; Yeaton et al., 2020) have shown the importance of changes in such body parameters to modify aerodynamic forces. Though these studies provide an understanding of how the animal's body interacts with the surrounding fluid medium to enable gliding, it is unclear if some or all changes in body shape and size are actively undertaken by the animal or are a passive effect of the animal's body experiencing external forces of fluid flow. For example, the wings of mammalian gliders and flying lizards change camber (a measure of wing curvature) during the glide. It is unclear whether camber changes are induced by the animal or are a consequence of the material properties of the wing under aerodynamic load, or both. A similar argument holds for the gliding pose undertaken by ants, frogs, and geckos, which warrants further investigation.

#### Body control using tail

Tailed terrestrial gliders undergo changes in body orientation during gliding that are often accompanied by rapid tail movement, suggesting a possible mechanism for controlling body orientation. Moreover, tails of gliding animals can be up to 1.5 times their body length, increasing the magnitude of inertial and aerodynamic effects on body orientation and ultimately aerodynamic force generation. Modeling has suggested that an active tail is beneficial for pitch stability in flying lizards and flying squirrels (Clark et al., 2021; Zhao et al., 2019), but observations in live animals are required to corroborate this finding. Overall, the aerodynamic versus inertial contribution of the tail towards body control requires further exploration.

#### Sensing

Few studies have investigated sensing in non-flapping flyers. The vision of flying snakes has been characterized preliminarily (Zamore et al., 2020), and vision-based models have been used to describe the gliding trajectory of flying lizards (Khandelwal and Hedrick, 2020). There is also evidence of multiple sensory modalities in use during gliding for some species. For instance, the presence of mechanoreceptor sensilla in the patagia has been found in *Gekko kuhli*, and these might be used for airflow sensing (Russell et al., 2001). Differences in the vestibular system have been found in gliding and non-gliding lizards and snakes (Boistel et al., 2011). Overall, the role of sensing and control in non-flapping gliding animals is greatly understudied compared to that in flapping flyers.

### Flapping flyers

#### Sensing

The use of upward drafts for soaring is well-demonstrated in seabirds and albatrosses. Kempton and colleagues suggest that Manx shearwater can use optical flow to extract wind direction information relative to their average heading to optimally control their soaring behavior (Kempton et al., 2022). This finding further expands the scope of vision use in birds to include airflow sensing. However, it remains unclear how birds identify upward drafts; it is likely that a combination of air and ground temperature, humidity, surrounding bird movement, and other factors inform the bird about the presence/absence of upward drafts. A combination of on-body sensing and motion capture could be used to identify the factors used by the bird to identify upward drafts.

#### Tail use for drag reduction

The use of the tail as a drag-reduction device in barn owls opens a new avenue of hypothesis testing in other birds that use gliding (Song et al., 2022). Moreover, it warrants a comparative approach between adept gliders like the albatross that use gliding for extended durations of time, compared to birds like swifts that use it intermittently. The presence of long, slender wings in albatross might impose a lower selective pressure for the use of tail for drag reduction during gliding, and hence a marginal contribution towards drag reduction compared to swifts, which have relatively stubbier wings.

## Future prospects

The Year at the Forefront studies highlighted here benefitted from advancements in engineering and technology, allowing them to collect more biologically relevant data, and consequently leading to new insights on the biomechanics, behavior, and sensing of gliding locomotion. Altogether, these studies further corroborate the concept of gliding as a sophisticated form of locomotion, and demonstrated the need for an interdisciplinary approach to gain a holistic understanding of gliding locomotion.

Future studies might blur the line between lab and field approaches, using advancements in engineering and technology to develop lab-like high-fidelity data collection tools and techniques that can be used in field settings. The development of new techniques will allow the study of gliding in biological systems that were previously extremely challenging. For example, new techniques will make it possible to study aerial gliding behavior in flying fish and squid in open waters, and even underwater gliding in a variety of aquatic animals, addressing the paucity of studies in each system.

As a research community, we are seeing the critical role of exploratory, discovery-based studies of non-model organisms in gliding locomotion (Clark et al., 2023). At first inspection, animals like wingless ants and salamanders show no hints of their abilities to glide, only discovered by researchers willing to ask, ‘what if?’. In the future, a combination of exploratory and hypothesis-driven studies will provide an opportunity to probe previously established hypotheses and generate new testable hypotheses with the availability of data that were not previously possible to collect.

Lastly, we are already witnessing the expansion of the field of gliding locomotion as it inspires new engineering efforts to enhance locomotion capabilities in robots, ranging from new wing designs to perching capabilities in micro-aerial vehicles. The future will see findings pertaining to gliding control, wing design, and wing materials translate to various engineering applications and the use of robots as a scientific tool to study gliding locomotion in animals.
